# Supplementary material for: Are carbon emissions trading and green financial instruments synergistic? -Comprehensive quantitative research based on content analysis
Source: PLoS One. 2024 Mar 7;19(3):e0298601. doi: 10.1371/journal.pone.0298601 (PMC10919601; doi:10.1371/journal.pone.0298601)
Supplement: S1 Data — (ZIP) [file pone.0298601.s001.zip › Data/地级市Z_F工作报告.zip!/╣π╬≈/╣≡┴╓╩╨/└┤╫╘╣1⁄2╓┌║┼í╛┬φ┐╦╩2╛▌═°í┐_2012.docx]

各位代表：

现在，我代表市人民政府向大会作工作报告，请予审议，并请各位政协委员和其他列席人员提出意见。

2011年工作回顾

过去的一年，我们在自治区党委、政府和市委的坚强领导下，牢牢把握科学发展主题和加快转变经济发展方式主线，从容应对复杂多变的外部环境和异常艰巨的改革发展任务，在“增投、扩量、稳速、提质、控价、保位”上下功夫，全市经济社会发展呈现增长较快、效益较好、结构优化、民生改善、物价趋稳的新格局，地区生产总值等6项经济指标超过千亿元，实现了“十二五”良好开局。

——— 地区生产总值突破千亿元大关后，继续保持强劲增长势头，预计达1336.13亿元，比上年增长12.2%。

——— 全社会固定资产投资突破千亿元大关，预计达1140.20亿元，增长28.9%，投资规模和重大项目建设数量再创新高。

——— 全部工业总产值突破千亿元后，继续快速增长，预计达1607.36亿元，增长35.0%。规模以上工业总产值突破千亿元大关，达1267.80亿元，增长41.7%；实现增加值突破400亿元，达404.58亿元，增长24.8%；主营业务收入突破千亿元大关，达1137.71亿元，增长38.6%；实现利税156.16亿元，增长90.8%。继食品、机械装备之后，电子信息、冶金有色2个产业跻身百亿元产业行列，我市百亿元产业达到4个。

——— 财政收入达141.94亿元，增长17.2%，高于预期4.2个百分点，其中一般预算收入80.75亿元，增长20.4%。

——— 金融机构存款余额超千亿元后，继续保持健康增长态势，达1554.9亿元，增长12.9%；贷款余额907.21亿元，增长14.9%。

——— 旅游总收入突破200亿元，达218.34亿元，增长29.7%，旅游业质量效益明显提升。

——— 农民人均纯收入突破6000元大关，达6325元，位居全区前列，增长15.3%，增幅创12年来最高；城镇居民人均可支配收入19882元，增长10.8%，超过全区平均水平。

——— 森林覆盖率69.05%，城市绿化覆盖率44.3%，均位居全区前列。

一年来，我们主要做了以下工作：

一、强力推进“四大建设”，发展基础更加坚实

完善重大项目联审联批制度，抓好项目筹融资和用地保障，以“四大建设”为主攻方向的重大项目建设取得新进展。

城市建设强力推进。新区建设与老城疏解提升并举，实施重点建设项目183项，完成投资250亿元，进一步改善提升了城市形象和品位。

交通能源水利基础设施建设再创佳绩。101个交通、能源、水利重点项目完成投资150亿元。贵广铁路、湘桂铁路扩能改造和兴安至桂林、阳朔至鹿寨、灌阳永安关至全州凤凰高速公路加速建设。桂林市防洪及漓江补水枢纽工程全面开工、进展顺利。我市风能基地建设处于全区领先地位。

园区建设成效显著。完成投资125亿元，增长38.8%。园区土地平整和基础设施建设加快推进，新增入园企业110家，开工亿元以上工业项目64项。

城乡风貌改造彰显特色。完成投资50亿元，主城区和12县老城临街建筑立面及道路街区绿化、美化、亮化改造提升。阳朔县兴坪镇、恭城瑶族自治县红岩村获“全国特色景观旅游名镇(村)”称号，兴安县兴安镇、灵川县大圩镇、龙胜各族自治县龙脊村等获“广西特色景观旅游名镇(村)”称号。

二、强力推动“两大改革”，发展优势明显提升

把规划建设桂林世界旅游城作为“两改”突破口，推动服务业转型升级。第三产业实现增加值467.58亿元，增长7.9%；三产税收占全部税收比重达54.4%，比上年提升2.9个百分点。

旅游综合改革试验区建设取得阶段性成果。桂林国家旅游综合改革试验区建设总体方案和建设发展规划纲要完成编制上报，《桂林世界旅游城概念规划》获自治区批准，我市列入“国家旅游产业用地管理改革试点城市”。罗山湖体育休闲产业园等30个桂林世界旅游城重点项目集中开工或竣工。旅游标准化建设取得新成效，阳朔县、兴安乐满地通过全国旅游标准化示范县、示范企业验收。全力推动漓江景区管理体制改革，加强旅游市场整顿监管，旅游服务质量进一步提高。旅游宣传营销范围不断拓展，国内外客源得到深度挖掘。接待游客2788.17万人次，增长24.1%，其中入境游客164.39万人次，增长10.6%。

国家服务业综合改革试点区域建设全面启动。积极争取自治区对桂林服务业综合改革试点区域建设的政策支持，制定出台开展国家服务业综合改革试点实施方案和配套文件，完成10个服务业专项规划编制及其项目库建设。启动象山区万福休闲旅游区、秀峰区桃花江社会化养老示范区、叠彩区城北体育文化消费示范区、阳朔县城乡互动生态旅游示范区等试点示范项目。推动旅游文化会展产业融合发展，成功举办第五届联合国世界旅游组织/亚太旅游协会旅游趋势与展望国际论坛、第三届桂林国际山水文化旅游节、第二届中国桂林国际旅游博览会、中国桂林创新创意文化节暨桂林国际动漫节、第二届名优产品展销会，“中国桂林国际”会展品牌影响力进一步扩大，中国桂林国际旅游博览会获中国会展业年度大奖，被评为2011年度中国最具影响力展览会50强；县区特色节事节庆文化旅游品牌效应增强，兴安米粉节被评为“2011年度中国十大品牌节庆”。交通运输、商贸物流、信息通讯、文体娱乐、家政服务等服务业发展迅速，房地产市场健康发展。实现社会消费品零售总额462.36亿元，增长18.1%。

三、强力调整产业结构，工业主导地位凸显

紧紧把握建设国家级信息化和工业化融合实验区机遇，着力打造千亿元产业和千亿元园区，加快改造提升传统产业，发展战略性新兴产业。全市产值超亿元企业新增67家,超10亿元企业新增5家。工业经济对全市地区生产总值增长贡献率达58.2%。

战略性新兴产业快速发展。制定实施《关于加快培育发展千亿元太阳能光伏产业的决定》，桂林光伏产业基地建设加快，尚科光伏、尚鼎新能源等13个项目竣工投产，光伏产业完成产值50亿元，增长10倍以上，以兴安光伏产业园为核心，高新区、临桂县等特色光伏产业园为支撑的桂林太阳能光伏产业带正在形成。工业投资高速增长，完成固定资产投资370.47亿元，增长30.6%；技改投资290.47亿元，增长34.4%，实施技改项目超1700项。桂林福达、啄木鸟医疗器械、思奇通讯、云尚动漫、资源县金紫山及龙胜南山风电场等强优企业和新兴产业快速发展，全市高新技术产业产值占工业总产值的比重超过31%，提升2个百分点。

园区经济强劲增长。出台《关于加快建设桂林国家高新技术开发区千亿元产业园区的决定》，高新区列入自治区“十二五”重点培育发展的千亿元园区，工业总产值突破200亿元，达到208亿元。推行园区管理体制改革，重点推进电子信息产业园、英才科技园、铁山工业园、生物医药产业园、汽车和橡胶产业园等特色园区建设，形成了以桂林国家高新区为龙头，苏桥经济开发区、西城经济开发区为重点，八里街工业园及各县工业集中区共同推进的园区发展格局。园区完成工业总产值750亿元，增长62.2%，实现工业增加值220亿元，增长41%，园区经济各项指标增幅均高于全市工业相应指标增幅。

“两化”融合试点全面启动。重点扶持两化融合示范试点企业200余家，信息技术更多应用于企业生产管理各环节，电子信息等支柱产业的自动化技术、信息化管理、数字化产品应用比例分别达76%、96%和65%。信息制造业产值超过100亿元，增长108%。网络带宽增长超过80%。

扶持企业发展扎实有效。实施中小企业“五十百千工程”，组建广西第一家中小企业信用担保协会，搭建国家开发银行桂林中小企业统贷平台，全年制造业贷款增长25%。

四、强力推进农业示范市建设，农村经济稳步发展

以创建循环农业示范市为契机，坚持大兴水利强基础，狠抓生产保供给，力促增收惠民生，着眼统筹添活力，“三农”工作继续保持全区领先水平。完成农林牧渔业总产值389.97亿元，增长5.4%，实现增加值244.70亿元，增长5.2%。

农田水利基本建设全面加强。完成水利水电投资25.76亿元，其中冬春水利建设完成投资17.5亿元，均列全区第一。39座重点小(一)型病险水库除险加固项目开工建设，水土保持和小流域治理成效显著。通过了全国节水型城市复查考核，作为广西唯一的全国水生态系统保护与修复试点城市通过验收。

着力推动现代农业加快发展。“吨粮万元田工程”扎实推进，建设吨粮万元田31万亩。粮食总产量190.07万吨，增长2.1%，我市被评为全区粮食生产先进单位。特色农业进一步增效，特色效益农业示范基地、示范点总面积达191.58万亩，水果、蔬菜、食用菌、毛竹、生猪、家禽等优势特色农产品生产持续增长，发展林下经济449万亩，万头猪场增至30个，兴安县获国家级“生猪调出大县”称号。以龙头企业、农民专业合作组织为载体的农业产业组织化程度进一步提高。循环农业进一步提质，“猪—沼—菜”“稻—灯—鱼—菇”等生态模式进一步推广。突出发展绿色、无公害食品，农产品质量安全合格率99%以上。乡村旅游农业快速发展，全年接待游客近600万人次，收入超过6亿元。阳朔、恭城率先在全区步入“全国农业休闲旅游示范县”行列。

新农村建设步伐加快。完成400个“普惠制”试点村建设，阳朔县“桂林山区新农村建设示范带”通过自治区验收。农村交通条件进一步改善，建制村客车通达率88.2%，新增130个建制村通水泥路，通畅率59.7%。扶贫工作成效突出，第三批“整村推进”82个村全部以优秀等级通过自治区验收。

县域经济发展迅速。预计12县实现地区生产总值925亿元，增长12.3%；规模以上工业总产值882.45亿元，增长49.4%；全社会固定资产投资901亿元，增长30.8%；财政收入68.68亿元，增长20.6%。临桂、阳朔获广西2010年“科学发展十佳县”称号，资源县获广西“科学发展进步县”称号。圆满成功举办庆祝龙胜各族自治县成立60周年活动。

五、强力拓展城市空间，城乡建设加快推进

围绕建设特大城市目标，拉大城市框架，拓展城市空间，完善市政设施，提升城市品位。完成固定资产投资1040.22亿元，增长26.5%；全市城镇化率40.2%，提升1.3个百分点。

桂林世界旅游城建设拉开帷幕。2011年是桂林世界旅游城建设启动、临桂新区建设实现全面突破的一年，投资规模大、开竣工项目多、涵盖领域广。实施重大项目58项，完成投资32.8亿元，涉及文化、科教、工业、医疗、道路、绿化、市政和其他重大基础设施等领域。核心区“五纵三横三环”路网基本成形，临桂大道等5条道路完工；创业大厦、“一院两馆”等多个标志性建筑封顶或接近封顶；防洪排涝及湖塘水系景观工程、中心公园等公益性项目全面建设；国际旅游接待中心、学校、医院、金融、商务等一批配套服务项目启动；原住民安置加快推进。

老城疏解改造提升加速。完成《桂林市2010—2020年城市总体规划》修编上报，市县乡土地利用总体规划全部通过审批。“三桥十路”“1212工程”继续推进，西二环路、阳江路、滨江北路、建干北路、芳华路等城市道路加快建设，万福东路和龙门大桥完成开工前准备，连通东西、贯穿老城的交通网络新格局正在形成。两江四湖(二期)工程及南溪河、小东江环境综合治理项目进展顺利。城北汽车客运站启用，城市主要节点改造提升工程完成。秀峰区琴潭旅游文化休闲园、叠彩区商贸物流园、象山区休闲旅游园、高新七星区产业园、雁山区科教园等特色园区建设稳步推进。“城乡清洁工程”十大专项整治深入开展。县城老城改造、新区建设步伐加快，城乡面貌进一步改善。

六、强力推进环境保护和节能减排，生态建设成效显著

以科学保护漓江、强化节能减排为重点，突出抓好生态文明建设，环境质量继续保持全国先进水平，“城考”连续17年名列全区第一，“国家环境保护模范城市”通过自治区复核验收预评估。

依法科学保护漓江取得新进展。《桂林漓江风景名胜区总体规划》《广西桂林会仙喀斯特国家湿地公园总体规划》通过国家评审，深入调研、主动配合、大力支持、积极争取自治区出台了《广西壮族自治区漓江流域生态环境保护条例》，实施科学保护漓江六大工程初见成效。

城乡生态建设持续有力推进。深入开展“绿满八桂”造林绿化工程，我市被评为全区“绿满八桂”造林绿化先进单位，获全区森林防火目标管理一等奖。大力推进生态县、乡(镇)、村建设，新增自治区级生态乡镇20个，生态村16个，市级生态村300多个。可建沼气池入户率78.91%，继续位居全区前列。严格实行耕地保护目标责任制，耕地保护考核连续四年全区第一。水土保持和地质灾害防治进一步加强。

节能减排工作取得显著成效。全市万元生产总值能耗下降3.2%，规模以上万元工业增加值能耗下降12.4%，化学需氧量、二氧化硫、氨氮和氮氧化物减排均控制在自治区许可范围。建筑节能超额完成自治区下达任务。公共机构水、电、油等各项消耗指标下降3%，城市污水集中处理率90.46%，生活垃圾无害化处理率100%。12县全部完成污水处理工程建设。燕京啤酒(桂林漓泉)股份有限公司成为广西第一家通过验收的工业循环经济示范企业。

七、强力推进改革创新，发展动力持续增强

坚持深化改革，扩大开放，推进科技进步与创新，以开放促创新，以创新促发展。

重点领域改革继续深化。稳妥推进“扩权强县”及统筹城乡综合配套改革。统计方法制度改革取得新成效，统计“四大工程”建设取得新进展。集体林权制度主体改革基本完成。医药卫生体制改革稳步推进，兴安县、永福县基层医疗机构人事分配制度改革成为自治区试点并得到推广，新型农村合作医疗保障覆盖全市所有1654个行政村，参合农民367.68万人，参合率97.87%，超过自治区7.87个百分点，政府举办的基层医疗卫生机构实现了国家基本药物制度和综合改革全覆盖，10类基本公共卫生服务项目实施率达100%，实施防治艾滋病攻坚工程、母婴健康“一免两补”等自治区自选医改项目成效显著。科技、教育、劳动保障、国企改革、民营经济等领域改革深入推进。

财政金融机制创新稳步推进。狠抓财源建设，加强财税监管，财政收支结构继续优化，对重点产业、重大项目扶持力度增强。全市民生保障支出增长35.9%，资金得到较好保障。国库集中支付改革全面铺开，政府采购工作不断完善。金融对经济社会发展的支撑力增强，国海证券借壳ST集琦正式挂牌上市并落户桂林，成为注册地在广西的唯一上市券商，我市年度企业上市和培育完成目标任务位列全区绩效考评第一；桂林银行作为广西地方银行率先在区外开设金融机构，华夏银行等金融机构落户桂林，融资性担保机构数量位居全区第二。

开放合作领域全方位拓展。主动融入国际国内多区域合作，加强与欧美、日韩、东盟、港澳台等国家和地区经济文化交流。深入开展大招商、大服务、大兑现活动,新签市外境内项目519个,新签项目总投资466.66亿元，增长20.8%；实施市外境内本年度到位资金项目912个，到位资金491.56亿元，增长31.4%，其中区外到位资金448.70亿元；实际利用外资3.67亿美元，增长46.7%，引进内外资总量均居全区前三位。实现外贸进出口总值9.57亿美元，增长5.4%。

科技创新能力深层次提升。顺利完成第四轮创新计划，全面启动第五轮创新计划，成功培育自治区级企业技术中心3家，获自治区科技奖23项，完成专利申请1767件，专利授权896件，每万人发明专利拥有量位居广西首位。争取国家、自治区科技项目496个，带动全社会科技投入18.9亿元。累计有8个县列入国家科技富民强县试点。

八、强力保障和改善民生，促进社会和谐发展

社会事业、社会保障、社会管理创新进一步加强。累计投入资金42.83亿元，市政府10项为民办实事工程年度任务圆满完成，改革发展成果进一步惠及人民群众。

城乡统筹就业全面推进。就业再就业及农村劳动力转移就业技能培训力度加大，全年新增城镇就业人数7.49万人，实现农村劳动力转移就业9.71万人，城镇登记失业率3.91%，控制在年度目标4.5%以内。

社会保障体系逐步完善。五项社会保险参保人数累计达242.14万人，基金征缴总额39.81亿元，处于全区领先水平。发放城乡低保资金2.95亿元，惠及低保对象344.61万人次。保障性安居工程项目开工率100%，新增保障性住房25173套(户)，完成自治区下达任务的107.3%，4033户城镇低收入住房困难家庭搬进新居，廉租住房租赁补贴惠及1.51万户。农村危房改造开工率100%，实际完成1.9万户。

物价上涨得到有效遏制。建立完善价格调控体制机制和目标管理责任制，率先在全区建立社会保障和救助标准与物价上涨挂钩联动机制，采取临时价格干预措施，动用价格调节基金600多万元，向1.8万名低收入居民发放了临时价格补贴；财政补贴1800多万元，及时向市场投放平价猪肉、油、米、燃气，居民消费价格涨幅控制在5.8%。

教育事业优先发展。全面实施国家和广西中长期教育改革发展规划纲要，颁布实施《桂林市教育布局调整规划(2011—2020年)》，各级财政对教育投入持续增长。学前教育三年行动计划全面实施，中小学校舍安全工程开工率位居全区前列，我市获“自治区职业教育攻坚先进市”，全州、灵川获“自治区职业教育攻坚先进县”，市职业教育中心学校进入首批国家中职教育改革示范校行列。县乡(镇)村三级联动(大学)生源地信用助学贷款贷后管理模式在全区率先推行，全年发放各种资助金1.9亿余元，受助贫困生15.6万人次。桂林各高校建设发展取得新成绩，为地方经济社会发展提供了人才和智力支持。

文化体育事业成果丰硕。未成年人思想道德建设成效显著，全国文明城市创建工作扎实开展。通过政府倡导、社会支持、百姓参与、媒体互动，“百姓大舞台”不断创新完善，得到中央、自治区充分肯定。“百姓大讲坛”“读书月”“漓江之声”“周末大家乐”及红歌演唱比赛等活动深受群众欢迎。文艺创作精品不断涌现，在第六届广西文艺创作铜鼓奖评比中，获奖数量继续领先；现代桂剧《何香凝》在全区成功巡演。《桂林市志》获广西地方志优秀成果一等奖。公共文化服务体系建设扎实推进，建成乡镇综合文化站24个、村级公共服务中心91个、农家书屋174家，并向社会免费开放。历史文化名城保护成果丰硕，全市第三次全国文物普查工作圆满完成，全州县贡陂堰荣获“第三次全国文物普查百大新发现奖”，龙胜龙脊壮族生态博物馆列入国家文物局首批公布的5个“全国生态(社区)博物馆示范点”。靖江王府及王陵、甑皮岩国家考古遗址公园保护建设工程全面启动，八路军桂林办事处纪念馆新陈列馆封顶。全民健身活动广泛开展，参加自治区第十二届运动会金牌、奖牌数和总分均获第三，成功举办首届桂林国际马拉松(半程)邀请赛等大型体育活动。

人口和计划生育工作保持全区先进。全国诚信计生暨人口计生基层群众自治工作座谈会在我市召开，诚信计生模式在全国推广。村、居委诚信计生推开面分别达93%、92%。建成387个“幸福家园”村级综合服务平台，人口计生优质服务水平不断提高。人口自然增长率低于7‰。

社会管理水平有效提升。全面开展大接访、大排查、大调解、大防控活动，信访维稳工作有新的提高。积极推进复合型警务机制改革，大力整治社会治安突出问题，严厉打击各类犯罪活动，人民群众安全感和满意度明显提升。加强应急管理，应对突发事件的处置能力不断提高。“五五”普法蝉联全国先进，法律援助工作“桂林经验”在全区推广。开展安全生产基层基础年活动，食品、药品监督管理得到加强。

一年来，我们坚持科学决策、民主决策，强化绩效管理，切实转变职能；坚持深入推进依法行政、政务公开和信息公开，政府执行力和公信力进一步提高；坚持重大事项向市人大及其常委会报告、向市政协通报制度，人大代表议案建议和政协提案办结率100%。广泛听取各民主党派、工商联、无党派人士意见，支持工会、共青团、妇联等群团组织发挥作用，凝聚各方智慧，共同推动我市经济社会平稳较快发展。国防教育、国防后备力量建设得到加强。民族、宗教、外事、侨务、接待、人事、编制、档案、保密、审计、新闻出版、机关事务、供销、气象、防震减灾、发展研究、社会科学、妇女儿童、老龄、残疾人等事业不断取得新进步。中直、区直驻桂单位不断取得新成绩。

一年来，我市相继荣获“国家科技进步先进市”“全国绿色模范城市”“全国十佳绿色城市”“全国法制宣传教育先进城市”称号，实现“全区双拥模范城”八连冠，获得“2011中国特色休闲城市——— 最美休闲城市”“最中国文化名城”等荣誉。过去一年所取得的成就和荣誉，使我市在全面建设小康社会征程上又迈出坚实一步，在富民强市新跨越道路上越走越宽广！

一年来，我们深切体会到：必须始终坚持以科学发展观为统领，加快发展方式转变，推动经济社会又好又快发展；必须始终坚持以项目建设为抓手，突出交通、产业、园区发展优先，持续保持投资强度，通过大项目、大投入带动大发展；必须始终坚持以改革开放为动力，进一步解放思想，先行先试，从根本上破除制约发展的体制机制障碍，加强自主创新和区域合作，增强发展活力；必须始终坚持经济建设和生态文明建设协调发展，切实节约资源和保护环境，实现人与自然和谐发展；必须始终坚持以人为本，切实保障和改善民生，促进社会和谐稳定，让人民群众共享发展成果。

各位代表，在十分复杂多变的国内外经济形势下，取得这些成绩实为不易。这是自治区党委、政府和市委总揽全局、正确领导的结果，是市人大、市政协大力监督和支持的结果，是全市上下同心同德、团结奋斗的结果。在此，我代表市人民政府，向奋斗在各条战线的全市广大干部群众及外地来桂工作者，向给予我们支持的人大代表、政协委员、各民主党派、工商联和社会各界人士，向中央和自治区各驻桂单位，驻桂解放军、武警官兵，向所有关心和支持桂林发展的朋友们，表示崇高的敬意和由衷的感谢！

在总结成绩的同时，我们也清醒地认识到，我市经济社会发展还存在不少困难和问题：受多重因素制约，我市经济增长的下行压力和物价上涨的压力并存；受资金、能源、用地等要素约束，保持投资较快增长难度增大，高铁、高速公路推进面临着政策调整带来的资金不足和开工不足等困难；部分企业受电力紧张、融资困难、原材料要素成本提高等影响，产能未能充分发挥，生产经营面临一些困难；由于国家采取新的统计制度，规模工业企业数量相对减少，规模工业培育工作任务艰巨；节能减排任务艰巨；涨价因素较多，保持物价总水平基本稳定难度增加；保障和改善民生、新一轮扶贫开发任务艰巨。对上述问题，我们一定要迎难而上，认真加以解决。

2012年工作总体要求和目标任务

今年是实施“十二五”规划承上启下的重要一年。自治区第十次党代会提出“翻两番、跨两步、三提高”的宏伟目标和加快建设“五区”的战略任务，以及市委四届二次全会提出实施“五大工程”的要求，为我们明确了奋斗目标和工作方向。我们一定要凝聚全市各族人民的智慧和力量，在加快实现富民强市新跨越的伟大实践中再创佳绩。

2012年政府工作总体要求是：全面贯彻党的十七大和十七届五中、六中全会以及中央经济工作会议、自治区第十次党代会、自治区经济工作会议和自治区十一届人大五次会议精神，紧紧围绕市第四次党代会、市四届人大一次会议、市委四届二次全会提出的战略部署，以科学发展为主题，以加快转变经济发展方式为主线，坚持走农业稳市、文化立市、旅游兴市、工业强市之路，牢牢把握“两改一融合一示范”机遇，坚持稳中求进、好中求快，深入推进经济结构战略性调整，全面实施项目建设、改革创新、生态建设、民生建设、文化建设工程，加快培育千亿元产业和千亿元园区，全力推进桂林世界旅游城建设，促进富民强市新跨越取得新进展，以优异成绩迎接党的十八大胜利召开。

今年经济社会发展主要预期目标是：地区生产总值增长11%，财政收入增长15%，全社会固定资产投资增长20%，规模以上工业增加值增长18%，万元地区生产总值能耗、化学需氧量排放量、二氧化硫排放量、氨氮排放量、氮氧化物排放量均控制在自治区下达指标内，城镇居民人均可支配收入增长11%，农民人均纯收入增长12%，城镇登记失业率控制在4.6%以内，人口自然增长率控制在9‰以内，居民消费价格总水平涨幅控制在自治区下达的指标以内。

各位代表，今年是我市各级领导班子完成换届后全面履职的第一年。随着我市发展基础的进一步夯实，随着我市区位交通优势、人才智力优势、政策叠加优势、金融支撑优势、产业发展优势、生态环境优势的日趋凸显，我们面临着加快发展的难得机遇，面临着赶超跨越的迫切需求，面临着建设桂林美好未来的历史使命。站在新的起点上，勤劳智慧的桂林人民将以更加宽阔的视野展望未来，将以更加创新的思维谋划发展蓝图，将以更加昂扬的激情加快建设我们的家园。只要我们切实增强危机意识、忧患意识、风险意识、责任意识，把困难估计得更充分一些，把应对措施准备得更周全一些，解放思想，赶超跨越，就一定能够保持全市经济社会发展的良好势头，谱写桂林富民强市的新篇章！

2012年工作部署

要实现今年的目标任务，必须紧紧抓住“两改一融合一示范”的重大机遇，全面实施“五大工程”，推动富民强市新跨越取得新成果。一是实施项目建设工程。要继续把投资和项目作为全市经济社会发展的重要拉动力，摆在重要位置，围绕经济社会发展各个方面统筹推进一批事关全市发展大局的产业、城建、基础设施和社会事业项目，加快实施今年第一批重点项目642项，完成投资600亿元以上，力争全社会固定资产投资达1368亿元、增长20%以上，为经济快速发展提供支撑和基础。二是实施改革创新工程。改革是发展的强大动力，要以更大的决心和气力加快“两改一融合一示范”推进步伐，力求在重点领域和关键环节改革上取得新突破，以改革增活力促发展。三是实施生态建设工程。牢固树立发展是政绩，保护良好生态环境更是政绩的理念，切实抓好科学保护漓江、森林覆盖及城市绿化工作，切实抓好江河源头、自然保护区、湿地、风景名胜区等保护利用，既要确保加快经济发展，更要确保创造良好的自然生态和人居环境。四是实施民生建设工程。以人为本，民生为重，突出抓好群众就业、增收、保障、安居以及新一轮扶贫攻坚工作，切实解决人民群众最关心、最直接、最现实的利益问题，让广大人民群众充分享受改革发展成果。五是实施文化建设工程。充分发挥桂林国家历史文化名城优势，以高度的文化自觉、坚定的文化自信、更大的文化作为，大力发展文化事业，加快发展文化产业，推动文化立市建设，努力走出一条具有桂林特色的文化改革发展道路，推动文化大发展大繁荣。

要重点抓好以下十个方面工作：

一、加快国家级信息化和工业化融合试验区建设，全面推动工业企业转型升级

以“两化”融合为契机，以推动转型升级为主线，以培育千亿元产业和打造千亿元园区为重点，加快改造提升传统产业，加快发展战略性新兴产业和园区经济，大力实施“抓大壮小扶微”工程，实现工业新跨越。力争全部工业总产值超2000亿元，增长23%以上，规模以上工业总产值超1500亿元，增长25%以上，规模以上工业增加值增长18%。

加快培育发展“468”产业体系。制定实施“468”重点产业扶持发展政策，围绕优势企业和优势产品，推动产业链向高附加值两端延伸，力争新增2个超100亿元产业。促进产业集群式发展，加快建设高新区创意产业园和大学科技园、苏桥经济开发区客车产业园和橡胶产业园、兴安工业集中区光伏产业园、西城经济开发区高端装备制造产业园和荔浦工业集中区衣架产业园、全州工业集中区湘山酒产业园等一批集中度高、关联度大、竞争力强、主导产业产值占比超过50%的特色产业园。

加快发展千亿元产业和千亿元园区。加快建设桂林光伏产业基地，推进尚鼎新能源铸锭切片项目(二期)等重点项目建设，扶持吉阳新能源、众阳光伏等重点光伏企业生产。加快高新区发展步伐，创新体制机制，鼓励发展“一区多园”的建设模式，进一步拓展发展空间，加快发展高端装备制造、电子信息、生物医药等三大支柱产业，培育发展新材料、新能源、创新创意等三大新兴产业，力争高新区工业总产值达260亿元。

大力推进工业项目建设。以投资结构的调整推进产业结构的优化升级，实施单项投资超500万元的工业项目500项以上，重点推进投资强度大、拉动作用强、技术含量高、市场前景广的投资超亿元工业项目50项。加快重大项目落地、开工、建设、竣工、达产。开工建设香港溢达、立晶光电等15个超亿元项目；加快推进电科院电工电子新材料产业基地、三金药业公司现代中药产业化工程等20个超亿元续建项目；突出抓好中橡桂林高等级子午线轮胎产业化项目(一期)、客车发展公司年产2.5万辆轻型客车搬迁改造等15个超亿元项目竣工投产；大力推进桂林福达汽车转向节生产线技术改造、桂林机床产业园等项目前期工作。进一步加大工业招商引资力度,引进一批名优企业、强优项目入驻。

深入实施“抓大壮小扶微”工程。推进实施中小企业“五十百千”工程，推动中小微企业与大企业建立配套协作关系，引导企业通过加强项目建设、技术改造、联合重组、企业管理等途径做大规模。实施十亿元企业、亿元企业、小企业上规模培育计划，力争新增10亿元以上企业2家；新增亿元以上企业30家，总量达到300家以上；新增及提升上规模企业60家，总量达到680家。

加快“两化”深度融合。推进云计算服务、环保节能监测、电子产品稳定性试验等“两化”融合服务和支撑平台建设，完成信息基础设施建设投资17亿元。突出汽车、机械、医药等重点产业及节能减排信息化应用，重点培育做强骨干软件企业。

着力提高自主创新能力。深入实施创新计划，加快构建创新体系，推进自治区级以上重点实验室(培育基地)、工程技术中心、检测中心、企业技术中心建设，支持建设特种轮胎及装备制造、微波与光通信等国家特色产业基地。加快完善科技成果转化体系，重点建设新兴产业研发孵化平台、多元化科技创新公共服务平台。大力实施知识产权战略，支持企业申报专利、争创驰名商标和品牌。围绕重点产业、战略性新兴产业开展核心关键技术攻关，加大对数控机床、中药新药等自治区千亿元产业重大科技攻关。

二、加快推进“两大改革”，推动现代服务业加快发展

以桂林世界旅游城建设为突破口，全力推进国家旅游综合改革试验区和服务业综合改革试点区域建设。实现第三产业增加值增长9.5%。

加快推进国家旅游综合改革试验区建设。着力打造大桂林国际旅游目的地和桂林旅游集散地，实现旅游接待人数增长10%、旅游总收入增长25%。力争《桂林国家旅游综合改革试验区建设发展规划纲要(2011—2020年)》得到国家批复。加快以桂林世界旅游城为重点的旅游产业项目建设，着力推进万福休闲旅游区、桃花江旅游度假区、罗山湖体育休闲产业园、国家森林公园、桂林国际旅游商品批发城改扩建、桂林国际足球产业园以及中华桂林航天城、兴安灵渠、龙胜龙脊、恭城山寨水寨等一批旅游产业项目建设，加紧会仙湿地公园前期工作。加快旅游交通网络、高端酒店等旅游配套设施建设。加快旅游产业转型升级，培育旅游新的增长极，促进旅游产业与文化产业等融合发展，着力打造四条旅游发展轴带、三大特色产品、三大水陆游线、七大旅游品牌，打造一批特色景观旅游名镇(村)和观光休闲农业示范点。加快旅游体制机制改革创新，重点完善漓江管理体制及游览经营机制，推进漓江游览及各景区景点服务质量标准化管理。加快旅游市场开发，强化旅游市场营销，争取和支持开辟新航线。加快完善旅游公共信息服务平台，积极申报国家旅游信息化试点城市。办好第六届联合国世界旅游组织/亚太旅游协会旅游趋势与展望国际论坛等市县(区)旅游节事节庆活动。

加快推进国家服务业综合改革试点区域建设。力争自治区出台加快推进桂林国家服务业综合改革试点的意见。加快服务业发展政策、措施和考评体系的建立完善。加快服务业综合改革“五大示范区”建设，加快推进城北现代物流配送中心、临桂新区商贸物流园、国家级服务外包基地、互联网产业基地、康复养生养老基地等一批服务业项目建设，建立完善服务业重点企业培育名录，加大企业培优扶强力度，努力构建服务业“345”产业体系。启动社会化养老服务产业管理体制改革。精心办好第三届中国桂林国际旅游博览会等展会。

积极扩大消费需求。深入开展扩大城乡居民消费“510”活动和美食、汽车、旅游节事等主题促消费活动，进一步扩大消费需求。完善城乡商品流通网络，提升改造一批农贸市场，推进“万村千乡市场工程”，加强“农超对接”工作，引导大型连锁超市与农产品生产基地建立长期的产销联盟。

三、加快农业示范市建设，全面加强“三农”工作

以建设循环农业示范市、特色效益农业示范市、吨粮万元田工程示范市、乡村旅游农业示范市为载体，加快现代农业建设，确保增加农产品有效供给和农民稳定增收。力争第一产业增加值增长4.6%。

毫不放松抓好粮食生产。加强基本农田保护和高标准基本农田建设，大力发展超级稻、玉米、马铃薯等粮食作物，确保粮食播种面积稳定在556万亩以上、粮食总产191万吨以上。

大力提升特色效益农业。加快实施“四大流域”及西部山区现代农业发展规划，推进区域化布局、标准化生产、规模化种养，着力打造优质粮食、水果蔬菜、畜牧水产、竹木林产、中草药材五大产业链，创建一批农副产品知名品牌。狠抓“菜篮子”工程建设，建立健全“菜篮子”产品有效供给体系。积极打造乡村旅游精品线路，提升观光休闲农业发展水平。

大力发展林下经济。巩固林权制度改革成果，推进林业配套改革。因地制宜发展特色林下经济，新增林下经济面积90万亩，实现林下经济产值15亿元，增长30%。

加强水利建设。全面加快桂林市防洪及漓江补水枢纽工程建设，力争川江水库建成并投入使用，小溶江水库完成大坝工程量70%以上，斧子口水库实现大江截流。抓好小型病险水库除险加固、中小河流治理工程及县城防洪、大中型灌区节水改造、小型农田水利等工程建设，提高农业防灾抗灾能力。继续加强农村饮水安全工程建设，新解决20万农村人口饮水安全问题。

加强农业科技支撑能力和社会化服务体系建设。加快建立农业科技创新和推广体制机制，大力推广农业实用技术，深入实施百万农民科技培训工程。提高农业生产耕种收机械化水平，增强农机综合服务能力。加强农产品安全检验检测，逐步建立农产品质量安全追溯监控体系。完善产后服务和流通体系建设，推广农产品采后保鲜和商品化处理技术，大力培育专业化服务组织、农村经纪人，着力培育一批物流冷链和加工销售带动型龙头企业。

推进农业农村基础设施和新农村建设。加快农村水电增效扩容和电网升级改造。大力实施农村公路通畅和连通工程，建制村通畅率达68%，稳步提高建制村客车通达率。加快农村广播电视和信息网络建设。大力实施村庄综合整治和农村危旧房改造，推进农村小型污水垃圾处理设施、公共服务设施建设，努力建设农民幸福生活的美好家园。

积极拓展农民增收渠道。全面落实强农惠农政策，确保农民政策性增收。加快实施“万元增收工程”和“百村示范、千村推进”工程，创建一批一村一品示范村，打造一批“吨粮万元田”“万元片区”“万元增收村屯”和“10万元致富示范户”，稳定增加农民生产性收入。加大农村剩余劳动力转移就业培训力度，积极发展劳务经济，增加农民现金收入。

四、加快特大城市建设步伐，推动城镇化跨越发展

以建设特大城市为目标，加快中心城市、县城、特色乡镇建设，推进新型城镇化进程。实现固定资产投资1248亿元，增长20%，城镇化率达41.8%以上。

举全市之力推进桂林世界旅游城建设。把世界旅游城作为旅游综合改革试验区建设的重要载体和突破口，按照“一点三星三区”布局，加紧实施《桂林世界旅游城概念规划》，加快专项规划编制。建立世界旅游城项目库，加快项目建设进度。全年计划实施重大项目207项，总投资754.5亿元，年度完成投资155.6亿元，新开工32项，续建117项，竣工13项。推进路桥基础设施建设，建成临苏路、新中路等道路，开工建设321国道雁山至临桂段改线(新雁路)、临桂新区核心区8座桥梁等工程，加快推进山水大道、凤凰西路等14条续建道路建设，完成北区主干道路、经三路等8条道路前期工作，同步完善地下管线。推进配套服务设施建设，创业大厦、“一院两馆”工程基本完工，加快新城商务酒店、金融大厦、广电中心、桂林日报社传媒中心及新城国奥小区等项目建设，启动旅游综合服务中心、工人文化宫等项目前期工作。推进民生保障项目建设，全力推进大律、岭上等7个原住民安置项目，加快一批院校新校区项目建设，启动中心医院、市民公园、临桂客运枢纽项目。推进环境改造及生态建设，重点实施中心公园、防洪排涝及湖塘水系景观工程、垃圾清运系统等项目。推进产业项目建设，加快临苏产业带、万福休闲旅游区、城南物流园区等项目建设，争取更多公益性优质资源进驻新区，不断增强桂林世界旅游城发展后劲。

强力推进老城疏解提升。进一步加强城市道路、桥梁等基础设施建设，形成与新区有机链接、更加科学合理的路网布局。加快西二环路、万福东路、龙门大桥、福利路一期及湖塘路等项目建设，继续抓好城市公共交通专项规划编制、停车场站规划建设及重要交通节点改造提升。进一步加强城市环境工程建设，改善城市环境，提升城市品位，全力承办好第二届广西(桂林)园林园艺博览会，打造“一路三园”景观带；加快推进南溪河、小东江环境综合治理和漓江洲岛改造等城市水环境建设，启动城北水厂扩建工程；大力实施小区拆墙透绿、主要道路节点绿化美化亮化工程。进一步加强城市特色园区和街区建设，调整完善城区功能布局，加快推进秀峰区琴潭文化休闲广场、叠彩区滨江文化体育休闲观光带及抗战文化一条街、象山区万福休闲广场、高新七星区七星路特色一条街、雁山区一站式购物中心等项目建设。

加快县城和小城镇建设。进一步修编完善县城和小城镇总体规划。加快城镇控制性详细规划编制。继续加快全州、兴安、阳朔、平乐、灌阳、灵川等县新区建设，提高产业和人口集聚能力。实施城乡风貌改造四期工程，打造一批旅游功能完善、桂北特色鲜明的名镇名村。

提高城市管理水平。建立规范高效城市管理体制，深入实施市容提升工程，加快推进数字化城市管理系统建设。加大对城中村、城乡结合部违法建筑整治力度，加强市政道路管理和维护。进一步深化拓展“城乡清洁工程”，健全完善市容环境卫生长效管理机制。建成山口垃圾填埋场，完成餐厨垃圾、电子垃圾处理项目前期工作。

五、加快推进文化建设工程，着力提升城乡文化实力

深入实施文化建设五大工程，不断完善公共文化服务体系，促进文化大发展大繁荣，提升城市文化实力。

大力发展公益性文化事业。加大对公益性文化事业建设的投入，全面实施文化发展规划，积极申报国家公共文化服务体系示范项目。继续实施文化精神工程，加强社会主义核心价值体系建设，深入开展精神文明创建活动，提高市民文明素质。继续实施文化惠民工程，推进基层文化馆(站)、图书馆、村级公共服务中心建设，努力实现“农家书屋”覆盖全市所有行政村，推动农村电影公益放映服务。继续实施文化景观工程，加快“一院两馆”等标志性文化项目建设，推进靖江王府及王陵、甑皮岩国家考古遗址公园等文物保护单位保护工程，加强历史街区、特色街区、传统风貌、文物及非物质文化遗产保护。继续实施文化精品工程，进一步加强桂林特色文学、美术、戏曲、民族歌舞等文艺精品创作，提升“百姓大舞台”“百姓文化大讲坛”“读书月”“漓江之声”“周末大家乐”等文化品牌，统筹各级文化节庆活动，构建桂林特色文化节庆体系。

加快发展文化产业。全面实施文化产业发展规划，重点培育发展演艺娱乐、文化旅游、工艺美术、游戏动漫、印刷包装及文化设备制造、数字化服务、会展服务等产业，加快构建桂林特色文化产业体系，大力扶持《印象·刘三姐》、愚自乐园、临桂五通农民画等文化产业示范基地建设，培育“桂林演艺之都”“桂林动漫基地”等文化产业品牌。继续实施文化管理工程，培育现代文化市场体系，组建发展一批骨干文化企业；引导文化市场经营项目向规模化、连锁化、专业化、品牌化方向发展；推动文化事业单位、经营性文化单位改革改制。

六、坚持交通优先发展，加强交通和能源基础设施建设

以交通、能源为重点，大力加强基础设施建设，为经济社会发展提供有力支撑。

加强交通建设。力争湘桂铁路(桂林段)扩能改造工程竣工，推进贵广高速铁路建设。加快高速公路建设，实现兴安至桂林高速公路建成通车，推进阳朔至鹿寨、灌阳永安关至全州凤凰高速路建设，新开工桂林至三江(桂林段)高速公路，加快资源至兴安、灌阳经恭城至平乐、荔浦至玉林(桂林段)、桂海高速(桂林段)改扩建等高速公路项目前期工作，力争开工建设。加快推进资源至龙胜、兴安至阳朔等路网项目及斧子口水利枢纽淹没路段复建工程建设，开工建设322国道灵川绕城线、永福至鹿寨等路网工程。加强公路运输枢纽建设，全力做好桂林北客运枢纽工程、桂林西货运枢纽工程、城东及城南汽车客运站等项目前期工作。科学实施《桂林港总体规划》，逐步推进桂林中心港和阳朔、平乐、全州、永福小港码头项目建设，完成磨盘山码头游客服务中心建设并投入使用，力争阳朔龙头山、平乐印山旅游码头开工建设。配合推动桂林两江国际机场航站楼及站场配套设施扩建工程前期工作。

加强能源建设。新开工国电永福发电有限公司2×35万千瓦上大压小热电联产扩建项目。加快桂林风电基地建设，推进资源县金紫山风电场二期、龙胜南山风电场一期等续建项目，新开工龙胜南山风电场二期、全州天湖风电场一期、兴安源江风电场、恭城燕子山风电场等项目，做好恭城园石山及西岭、灌阳马头风电场等项目前期工作。全年风电项目计划完成投资21.3亿元，新增风电装机容量9.9万千瓦，总量达到14.85万千瓦。

七、加快生态文明示范市建设，不断提升可持续发展能力

坚持把生态建设和环境保护摆在更加突出位置，推进生态文明示范市建设，保护和发展桂林生态环境优势，全面提升可持续发展能力。

依法科学保护漓江。严格执行《广西壮族自治区漓江流域生态环境保护条例》，推动《漓江风景名胜区总体规划》尽快获批实施。加强自然山水生态修复、饮用水源和生物多样性保护。推动建立漓江生态环境保护补偿机制，继续实施漓江源头水源林保护、漓江两岸绿化美化、漓江两岸及水域环境保护、漓江两岸富民惠民、漓江水域管理、漓江风景区旅游产品策划等科学保护漓江六大工程。

推进生态文明建设。全面实施生态市建设规划，大力发展循环经济。扎实推进漓东公园、桂林园林植物园二期等重点公园绿地建设。开展大气PM2.5监测，改善空气质量。加强重金属污染治理，争取“国家生态园林城市”获得批复“国家环境保护模范城市”通过国家复核验收。加大生态县规划实施力度，大力开展生态乡(镇)、村建设。落实最严格的耕地保护制度，提高土地节约集约利用和矿产资源综合利用水平。

全面推进“绿满八桂”造林绿化工程。加强植树造林和植被保护，积极创建国家森林城市，力争完成造林面积40万亩。抓好大中型沼气池建设工作，新建农村沼气池1.1万座。

版）加强节能减排工作。完善节能减排目标责任制考核办法，扎实抓好重点领域节能和清洁生产，加快淘汰高耗能、高排放落后产能企业，创建一批清洁生产示范园区。深入开展企业节能低碳行动、绿色建筑行动和全民节能减排行动，推进可再生能源建筑应用示范城市建设。加强重点节能减排工程建设，完成市东区污水处理厂扩建、临桂新区污水处理厂、漓江河堤排水口改造、骝马山北巷片区截污工程等城市污水处理系统建设。

八、深化改革扩大开放，全面增强发展动力活力

以改革开放为赶超跨越的根本动力，深入实施改革创新工程，着力解决制约发展的体制机制问题，推进开放合作领域不断拓展、水平持续提高。

深化重点领域改革。继续抓好“扩权强县”，深化城区管理体制和统筹城乡综合配套改革。积极推进统计制度方法改革，抓好服务业统计改革试点工作。加强财税金融体制改革创新，健全中小企业信用担保体系。不断深化国有企业改革。推动农村土地承包和流转规范管理，促进农村土地适度规模经营。推进集体林权制度配套改革。继续深化行政管理体制改革，加快科技、教育、文化、卫生、体育等事业单位分类改革。

提高开放合作水平。积极参与第九届中国-东盟博览会、泛珠论坛和桂台经贸论坛，主动融入国际国内多区域合作，实施“招大引强”战略，扎实推进“央企入桂”“民企入桂”。大力实施“走出去”战略，引导企业开展境外投资和设立海外发展基地。加快国家和自治区科技兴贸创新基地建设，着力打造面向东盟的农产品出口基地。

九、千方百计抓好投融资工作，积极推进投融资结构优化

调整优化投融资结构，突出发展实体经济，继续抓好项目建设，加强财税金融支撑，提高招商引资水平，促进扩大民间投资，保持投资对经济发展的强劲拉动。

全面加强项目工作。加大财政支持力度，优先保障续建项目和收尾工程。抓好土地征储、供应工作，优先保障重点产业、民生工程和重大基础设施项目用地需求。完善重大项目联动协调、督查督办、考评问责机制，强化重大项目跟踪服务，完善联合审批制度，推行并联审批，加快项目建设和前期工作。推动市、部门及县(区)投资项目库建设，继续加强重大项目谋划和储备工作，保持投资工作的连续性。

进一步扩大融资。深化政银企合作，加快地方金融主体建设，大力发展小额贷款公司，规范发展融资性担保公司，完善贷款担保体系，加大对中小微型企业信贷支持。培育漓东科技金融中心。加快培育上市后备企业资源，推进企业上市融资和再融资。推进企业债券融资，争取国家核准发行企业债券12亿元以上。

进一步加大招商引资力度。以特色农业、高新技术产业、文化创意产业和现代服务业等领域为重点，创新招商方式，突出招大引强，拓宽招商渠道，优化投资环境，深化项目兑现。在去年目标任务的基础上，力争引进市外境内资金增长20%、实际利用外资增长20%。

进一步激活民间投资。落实促进民营经济发展的政策措施，鼓励和引导社会资本进入城市更新改造、基础设施建设、基础产业、社会事业、国企改制重组、金融服务等领域，力争民间投资增长40%以上。

进一步做好财税工作。认真落实积极财政政策，加强财源建设，强化税收征管，提高收入质量。优化财政支出结构，加大民生领域投入，优先保证保障性安居工程、农业农村基础设施、社会公益事业、生态环保、新兴产业发展等投入，加大对光伏等新兴产业、工业园区及自主创新扶持力度。严格控制一般性支出，进一步提高政府预算透明度。

十、扎实抓好民生工作，加快和谐社会建设

保障和改善民生是政府的责任，是一切工作的出发点和落脚点，要进一步加大投入，加强各项民生工作，坚持社会事业协调发展，大力推进社会管理创新，维护和保持社会和谐稳定。

千方百计扩大就业规模。重点拓展服务业、小微型企业就业渠道，深入推进“全民创业”计划，积极帮助高校毕业生、农民工、就业困难人员等重点群体实现就业。新增城镇就业6.2万人以上，新增农村劳动力转移就业8.5万人以上，下岗失业人员再就业1.5万人。

健全覆盖城乡社会保障体系。推进新型农村社会养老保险试点，力争新农保和城镇居民社会养老保险实现城乡全覆盖。全市职工基本养老保险、失业保险、基本医疗、工伤、生育保险参保人数分别达到39.2万人、24万人、117万人、34万人、27万人。进一步提高城乡低保和社会救助水平，完善社会救助和保障标准与物价上涨挂钩联动机制。加大保障性住房建设和廉租房租赁补贴力度，惠及更多的中低收入住房困难家庭，促进房地产市场平稳健康发展。

优先发展教育事业。落实教育经费法定增长机制，加快实施教育布局结构调整规划，重点推进教育“双十工程”。深入推进学前教育三年行动计划，巩固提高“两基”和职业教育攻坚成果，推进创建自治区示范高中工作，争取完成聋哑学校建设搬迁。支持高等学校扩建和新院搬迁。

加快发展医疗卫生事业。深化医药卫生体制改革，继续提高新农合保障水平，抓好兴安县人民医院和永福县中医院的公立医院改革试点，加强基本公共卫生服务建设。强化医疗卫生机构监管，建立医疗纠纷处置和调解机制，健全重大疾病防控应急机制，推进基本公共卫生服务逐步均等化。

积极发展体育事业。深入开展全民健身运动，积极参加自治区第七届农民运动会，办好市第二届少数民族传统体育运动会、第二届桂林国际马拉松(半程)邀请赛、中国环桂林公路自行车赛、中日韩围棋世界冠军桂林争霸赛等赛事。

抓好人口和计划生育工作。实施诚信计生巩固提升扩面工程，县(区)基本实现诚信计生。抓好流动人口计划生育基本公共服务均等化、幸福家园建设、查处“两非”等重点工作。

做好物价调控工作。加强市场价格监督检查，建立完善价格预警机制、市场价格监管长效机制。切实增加农产品有效供给，增加大宗商品储备，建立健全调控和应急保供预案。

实施新一轮扶贫开发攻坚战。按照国家最新公布的2300元扶贫标准，加大对全市扶贫对象脱贫致富工作力度。构建专项扶贫、行业扶贫和社会扶贫“三位一体”的大扶贫格局，实行扶贫开发与农村最低生活保障“两项制度”有效衔接。科学编制扶贫特色优势产业规划，优先在贫困地区安排资源开发和深加工项目，培育壮大扶贫龙头企业和贫困地区特色优势产业，全力实施新一轮第一批132个整村推进扶贫开发，加强贫困地区基础设施及公共服务设施建设。

强化社会管理与创新。完善基层管理服务体系，加强城乡社区自治和服务组织建设。健全维护群众权益机制，完善社会稳定风险评估。抓好社会管理创新综合试点、社会管理信息化建设。加强对流动人口、农村留守人员及特殊人群的管理服务。全面开展“六五”普法，完善矛盾纠纷排查、调处、化解工作机制，积极预防和化解各种不稳定因素。深入推进平安建设，加强新形势下社会治安防控体系建设，严厉打击各类犯罪。切实加强道路交通安全、消防安全、安全生产、食品药品安全、环境安全以及应急管理等工作，增强人民群众安全感，确保社会和谐稳定。深入开展国防动员和国防教育，加强人民防空及国防后备力量建设，深化双拥模范城创建成果，巩固和发展军政军民团结。

继续实施惠民工程。筹措资金35亿元以上，办好以下十项惠民工程。

1.社保惠民工程。全面实现城乡居民社会养老保险、基本医疗保险全覆盖，其中2012年元月起实现新农村社会养老保险全覆盖。为全市6.3万城镇低保对象、31万农村低保对象发放最低生活保障补助，新建150个五保村。完善一线环卫工人工资增长机制，逐步提高一线环卫工人工资待遇。继续实施“阳光家园计划”项目，为3400名残疾人建立托养机构和居家托养服务。

2.卫生惠民工程。新型农村合作医疗筹资标准由每人230元提高到每人290元，财政对新型农村合作医疗补助由每人200元提高到每人240元。深入实施艾滋病攻坚工程、母婴健康“一免两补”幸福工程和地中海贫血防治计划。实施农村改厕项目，完成1.5万座改厕任务。加快农村医疗卫生服务体系建设，新建、改扩建23个县级医院、乡镇卫生院业务用房及污水处理、垃圾处理、配电设施等辅助设施。实施农村急救体系建设项目，改扩建5个县急救中心并购置设备。

3.教育惠民工程。积极筹措资金，扩大农村学前教育资源，实施农村义务教育学生营养改善；对农村义务教育阶段家庭经济困难寄宿生给予生活补助，对农村义务教育阶段学校校舍进行维修改造，发放普通高中国家助学金，对家庭贫困的大学新生给予资助，确保每一个孩子不因家庭困难而失学。推进贫困地区农村义务教育寄宿制学校和职业教育基础能力建设。加强校车安全管理。

4.强基惠民工程。继续实施行政村通水泥路或沥青路工程，修建一批村级道路。加大对小型农田水利重点县建设投入，完成中小河流治理和病险水库除险加固任务，逐步解决农村居民饮水安全问题。抓好库区移民新村建设。新建400个普惠制新村。实施1500个自然村(屯)道路硬化工程。

5.安居惠民工程。开工建设保障性住房1.73万套。以解决全市农村小学教师住房难为重点，加快推进公共租赁住房、廉租住房、经济适用住房、限价商品住房等建设。继续做好农村危房改造项目。抓好农村居民住房政策性保险工作。

6.土地整治惠民工程。按照自治区下达的计划,完成土地整理、中低产田改造、高标准农田示范等工程项目任务，按要求完成在贫困地区实施坡改梯和基本农田建设任务。

7.农补惠民工程。继续筹措资金用于粮食作物良种补贴、农资综合补贴、农机具购置补贴等项目。

8.文化计生惠民工程。建设131个村级公共服务中心，实施农村电影公益性放映、20户以下自然村通广播电视工程项目。实施免费孕前优生健康检查、村级人口计生健康服务室和新家庭文化书屋建设等项目。

9.政务服务惠民工程。推进政务服务、政务公开向基层延伸，新建30个乡镇(街道)政务服务中心。

10.生态惠民工程。实施“绿满八桂”工程、石漠化综合治理工程、森林生态效益补偿项目等，继续推进沼气池建设，实施村(屯)农村环境综合整治项目。继续推进南溪河、小东江综合治理项目。对芦笛路、建干路等14条街区道路进行推广使用新能源路灯节能改造。继续实施城北水厂二期扩建工程。完成第二届广西(桂林)园林园艺博览园建设工程。

各位代表，全面完成今年各项工作任务，对政府自身建设提出了更高要求。我们要牢记宗旨，为民施政。深入体察民情，认真倾听民意，使政府工作更加符合人民意愿。我们要忠于职守，务实勤政。完善政府绩效管理和评价机制，加强各级政务服务中心建设，力戒官僚主义和形式主义，形成干事创业的良好风气。我们要开拓奋进，依法行政。自觉接受市人大及其常委会的法律监督、工作监督和市政协的民主监督，认真听取各民主党派、工商联、无党派人士和各人民团体的意见，努力破除制约发展的体制机制性障碍，最大限度激发发展活力。我们要秉公用权，清廉从政。立身不忘做人之本、为政不移公仆之心、用权不谋一己之利，加强对权力运行的制约和监督，坚决纠正损害群众利益的不正之风。

各位代表，面对难得的发展机遇和光荣艰巨的发展任务，我们一定要更加紧密地团结在以胡锦涛同志为总书记的党中央周围，在自治区党委、政府和市委的正确领导下，牢记使命，恪尽职守，团结一致，开拓创新，锐意进取，为实现富民强市新跨越而努力奋斗，以优异的成绩迎接党的十八大胜利召开！
